# Using medicare claims to estimate risk-adjusted performance of Pennsylvania trauma centers

**DOI:** 10.1371/journal.pdig.0000263

**Published:** 2023-06-02

**Authors:** Alexis M. Zebrowski, Phillipe Loher, David G. Buckler, Isidore Rigoutsos, Brendan G. Carr, Douglas J. Wiebe

**Affiliations:** 1 Department of Emergency Medicine, Icahn School of Medicine at Mount Sinai, New York, New York, United States of America; 2 Department of Population Health Science and Policy, Icahn School of Medicine at Mount Sinai, New York, New York, United States of America; 3 Institute of Translational Epidemiology, Icahn School of Medicine at Mount Sinai, New York, New York, United States of America; 4 Computational Medicine Center, Sidney Kimmel Medical College, Thomas Jefferson University, Philadelphia, Pennsylvania, United States of America; 5 Department of Emergency Medicine, University of Michigan, Ann Arbor, Michigan, United States of America; 6 Department of Epidemiology, School of Public Health, University of Michigan, Ann Arbor, Michigan, United States of America; University of Manitoba, CANADA

## Abstract

Trauma centers use registry data to benchmark performance using a standardized risk adjustment model. Our objective was to utilize national claims to develop a risk adjustment model applicable across all hospitals, regardless of designation or registry participation. Patients from 2013–14 Pennsylvania Trauma Outcomes Study (PTOS) registry data were probabilistically matched to Medicare claims using demographic and injury characteristics. Pairwise comparisons established facility linkages and matching was then repeated within facilities to link records. Registry models were estimated using GLM and compared with five claims-based LASSO models: demographics, clinical characteristics, diagnosis codes, procedures codes, and combined demographics/clinical characteristics. Area under the curve and correlation with registry model probability of death were calculated for each linked and out-of-sample cohort. From 29 facilities, a cohort comprising 16,418 patients were linked between datasets. Patients were similarly distributed: median age 82 (PTOS IQR: 74–87 vs. Medicare IQR: 75–88); non-white 6.2% (PTOS) vs. 5.8% (Medicare). The registry model AUC was 0.86 (0.84–0.87). Diagnosis and procedure codes models performed poorest. The demographics/clinical characteristics model achieved an AUC = 0.84 (0.83–0.86) and Spearman = 0.62 with registry data. Claims data can be leveraged to create models that accurately measure the performance of hospitals that treat trauma patients.

## Author summary

We can leverage claims data to create models that accurately measure the performance of trauma centers while also accounting for patient case mix and injury severity. This represents a new way to benchmark trauma management at hospitals that treat trauma patients but are not accredited as trauma centers. Moreover, this approach can guide the development of regional models of care by using population level outcomes to encourage trauma centers, non-trauma center hospitals, and prehospital systems of care to optimize triage and transfer policies on regional survival.

## Introduction

Trauma is unplanned, time-sensitive, and carries a high societal burden of mortality and morbidity. The National Academy of Medicine recently highlighted the need for a national trauma system [1]. Over the past three decades, hospitalizations for injury have risen among those over age 65, with over three million injury-related Emergency Department (ED) visits and 55,000 deaths in 2017 alone [2–4]. The system of care developed around trauma—from ambulance services and emergency departments to intensive care units and rehabilitation centers—markedly reduces injury-related death and disability. Survival is significantly higher for seriously injured patients who are treated in Level I Trauma Centers [5–7]. Despite this, approximately two-thirds of patients are cared for in non-trauma centers, as are 70% of injured adults over 55 who receive hospital care [7–9]. Understanding the care of the trauma patient population, and identifying opportunities to improve trauma care at non-trauma centers is important for improving outcomes for the entire population of injured adults.

The trauma community has pioneered efforts to measure and benchmark trauma center performance through the Trauma Quality Improvement Program (TQIP). These efforts use a standardized risk adjustment model to benchmark trauma center performance [10]. Many states (43%) have trauma data registries [11], providing an indispensable resource for quality assurance and improvement initiatives. Data are often reported to a central credentialing agency, such as a state Department of Health or the American College of Surgeons, and are pooled to describe the experience of the state or nation in performance improvement initiatives. Facility level outcomes are benchmarked and compared in a de-identified manner across similar centers [10,12].

A key shortcoming of trauma registries is that only 13 states require non-trauma center hospitals to submit their data, limiting the ability to measure care delivered outside of the accredited trauma centers. The National Quality Forum recently released a framework for population based quality measurement for trauma care in the United States [13]. Enacting this will require a standardized and comprehensive approach that leverages compulsory data rather than a voluntary registry limited to specially credentialled hospitals.

The Centers for Medicare & Medicaid (CMS) are the largest purchaser of trauma care in the United States [14]. Claims data for age-eligible beneficiaries (65 years or older) are available uniformly at trauma and non-trauma centers, and contain information including patients’ injury mechanism and severity, physiology, comorbidities, and disposition. The objective of our study was to capitalize on these comprehensive data to develop a risk adjustment model that can be applied across trauma and non-trauma centers alike. This approach to measuring trauma outcomes and hospital performance aims to deliver a method of assessing trauma care quality, regardless of hospital designation or registry participation.

## Results

### Linking

**Fig 1:**
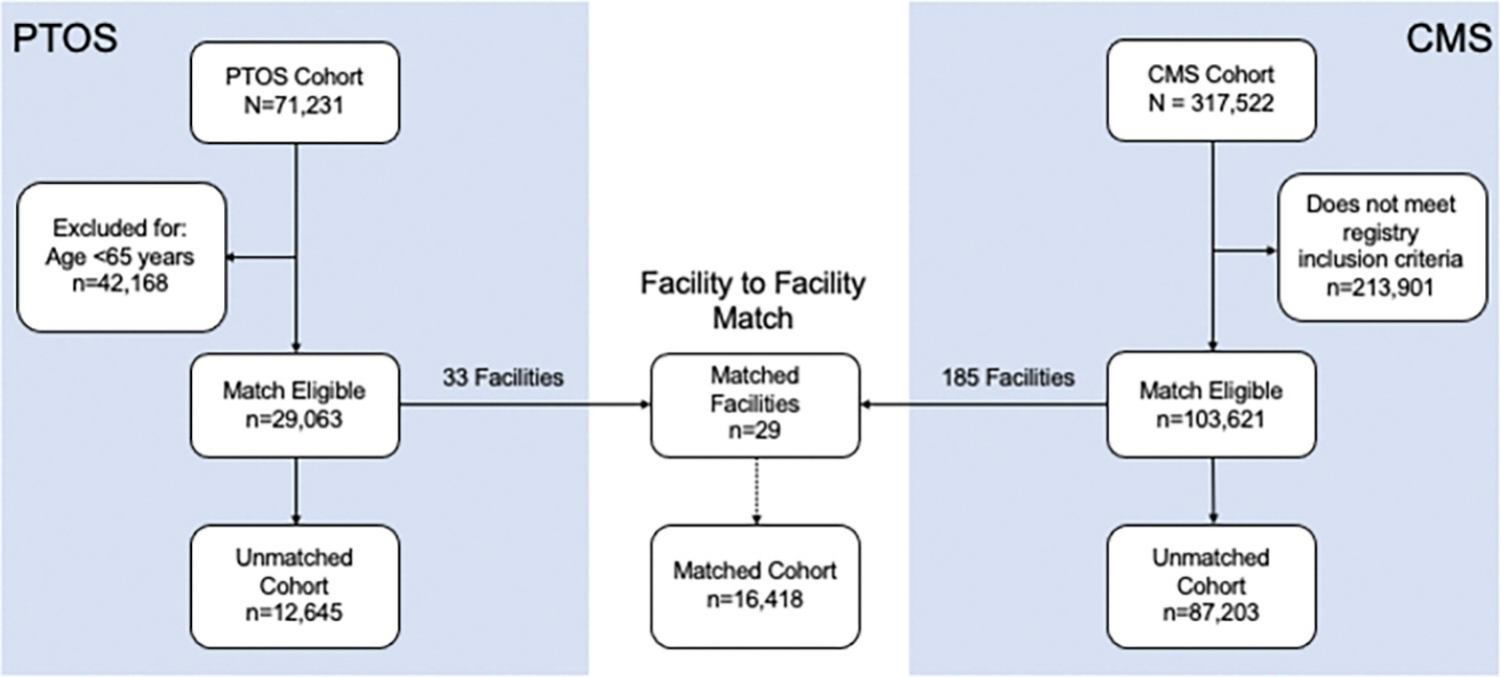
Cohort eligibility and matching between Pennsylvania Trauma Outcomes Study and Medicare Data, 2013–2014.

A total of 29,063 cases across 33 trauma centers were identified as eligible for linking in the PTOS data, while 103,621 possible cases across 185 centers were identified from the Medicare claims. Patient-to-Patient linking required a total of 6,105 facility-to-facility comparisons (**Fig 1**). The smaller number of cases in the PTOS data dominated the linking attempts creating a matched cohort comprising 16,418 cases (16,058 unique patients) from 29 facilities linked between PTOS and Medicare. These facilities included 11 level I, 13 level II, 2 level III, 1 level IV, and 3 dual accredited pediatric (level I or II) and adult (level I) trauma centers.

With regard to demographics, the patients were distributed similarly in both cohorts: median age 82 (IQR: 74–87) in PTOS versus 82 (IQR: 75–88) in Medicare; 6.2% non-white in PTOS versus 5.8% non-white in Medicare. The registry cohort comprised more instances of falls (n = 13,654, 83.2% compared to n = 12,134, 73.9% in claims data). Also, more registry patients had a max abbreviated injury score < 3 (n = 7,633, 46.5% versus n = 7,315, 44.6%). **Table 1** shows the demographic and injury characteristics of the linked and full cohorts.

**Table 1:** Characteristics of patients in the Pennsylvania Trauma Outcomes Study and Medicare data, 2013–2014.

Patient Characteristic, n (%)	Full PTOS Cohort* N = 29,063	Matched PTOS Cohort n = 16,418	Patient Characteristic, n (%)	Full Medicare Cohort* N = 103,621	Matched Medicare Cohort n = 16,418
Died	1,733 (5.96)	979 (5.96)	Died	3,417 (3.30)	979 (5.96)
Age in years, median (IQR)	81 (73–87)	82 (74–87)	Age in years, median (IQR)	83 (74–88)	82 (75–88)
Female	16,809 (57.84)	10,034 (61.12)	Female	66,761 (64.43)	10,065 (61.30)
Race			Race		
*White*	26,712 (91.91)	15,399 (93.79)	*White*	96,418 (93.05)	15,459 (94.16)
*Black*	1,389 (4.78)	696 (4.24)	*Black*	5,398 (5.21)	700 (4.26)
*Other/Unknown*	962 (3.32)	323 (1.96)	*Other/Unknown*	1,805 (1.74)	259 (1.58)
Mechanism			Mechanism		
*Fall*	23,045 (79.29)	13,654 (83.16)	*Fall*	71,327 (68.83)	12,134 (73.91)
*Motor Vehicle*	3,639 (12.52)	1,622 (9.88)	*Motor Vehicle*	2,709 (2.61)	1,504 (9.16)
*Fire/burn*	265 (0.91)	116 (0.71)	*Fire/burn*	616 (0.59)	100 (0.61)
*Pedal cyclist*, *other*	149 (0.51)	71 (0.43)	*Pedal cyclist*, *other*	161 (0.16)	64 (0.39)
*Other/Unknown*	1,965 (6.76)	955 (5.82)	*Other/Unknown*	28,808 (27.80)	2,616 (15.93)
Transfer	9,530 (32.79)	3,936 (23.97)	Transfer	5,126 (4.95)	700 (4.26)
Systolic BP, median (IQR)	149 (130–168)	150 (131–170)	Shock	284 (0.27)	36 (0.22)
GCS Motor, median (IQR)	6 (6–6)	6 (6–6)	Altered Mental Status and/or Impaired Sensorium	4,291 (4.14)	213 (1.30)
Pulse, median (IQR)	80 (70–93)	80 (70–92)	Tachycardia	1,140 (1.10)	317 (1.93)
Diabetes	7,838 (26.97)	28 (0.17)	Diabetes	29,070 (28.05)	4,408 (26.85)
PVD	1,041 (3.58)	131 (0.80)	PVD	8,899 (8.59)	1,285 (7.83)
Max Abbreviated Injury Score			Max Abbreviated Injury Score		
*0–1*	2,474 (8.51)	1,064 (6.48)	*0–1*	27,056 (26.11)	986 (6.01)
*2*	11,257 (38.73)	6,538 (39.82)	*2*	34,397 (33.20)	6,329 (38.55)
*3+*	15,149 (52.12)	8,785 (53.51)	*3+*	41,344 (39.90)	8,974 (54.66)
*Unknown*	183 (0.63)	31 (0.19)	*Unknown*	824 (0.80)	129 (0.79)
Injury Severity Score, median (IQR)	9 (5–13)	9 (5–13)	Injury Severity Score, median (IQR)	5 (2–9)	9 (4–16)

*Full cohorts contain only those registry patients or claims meeting eligibility criteria for the study (age > = 65, treated in Pennsylvania hospital in 2013 or 2014, documented trauma via ICD-9-CM code, and meeting registry eligibility criteria.)

For manual validation of the facility matching, 18 facilities volunteered to be identified in the PTOS registry. This allowed us to manually validate our automated facility matching and confirm that all 18 of these facilities were correctly paired between PTOS and Medicare datasets. For the remaining 11 facilities, trauma center designation was cross-referenced and matched in both the PTOS and Medicare data. Characteristics between the datasets appear similar with lower rates of transfers and higher prevalence of peripheral vascular disease (PVD) and diabetic comorbidities seen in the Medicare cohort (**Table 1**).

### Registry-based benchmarking

Using the linked PTOS registry data, an AUC of 0.86 (95% CI 0.84–0.87) was estimated for the GLM model and 0.85 (95% CI 0.83–0.86) for the LASSO model. Probability of death values were then generated from each model for each individual case: the two models agreed considerably (Pearson correlation = 0.98; Spearman rank coefficient = 0.93). Given the agreement in both AUC and probability of death correlations, and the need to handle large numbers of clinical attributes in the CMS dataset, we chose the LASSO model for all subsequent analyses. LASSO’s dimensionality reduction eliminated 41.5% of the predictor variables (S1 Table) with minimal impact to AUC.

### Model performance

The initial Medicare claims model included only beneficiary demographics: age, sex, and race/ethnicity. Using the linked PTOS-Medicare cases, the model predicted mortality with an AUC of 0.62 [95% CI 0.60–0.64]. The out-of-sample AUC was similar (**Table 2**). Comparison of the claims-based model and the registry model produced a Spearman rank correlation of 0.44 (**Table 2**). Using the linked data, procedure and diagnosis code models achieved an AUC of 0.86 (95% CI 0.84–0.87) and 0.95 (95% CI 0.94–0.96), respectively. The performance of the procedure-codes-only model showed a noticeable drop in performance in out-of-sample testing with an AUC of 0.76 (95% CI 0.75–0.77) vs. 0.91 (95% CI 0.90–0.92). The procedure and diagnosis codes model and the registry model showed virtually no correlation (**Table 2**). The final model, which adjusted for patient demographics and clinical risk factors, achieved an AUC of 0.84 (95% CI 0.83–0.86) for matched samples and 0.81 (95% CI 0.80–0.82) for out-of-sample records. The final model also had the strongest correlation with the registry model (0.62). Coefficients for variables in all claims-based models can be found in S2 Table which list all of the procedure and diagnosis codes that were used.

**Table 2:** Model performance diagnostics for Medicare claims models.

Data Source	Model	Matched Area Under the Curve (95% Confidence Interval)	Out-of-Sample* Area Under the Curve (95% Confidence Interval)	Spearman’s Correlation Coefficient (with Registry Model)
N/A	Control	0.50	0.50	0.00
PTOS**	Registry	0.85 (0.83–0.86)	0.88 (0.86–0.89)	1.00 (self)
Medicare	Demographics	0.62 (0.60–0.64)	0.60 (0.59–0.61)	0.44
	Demographics + Clinical Risk Factors	0.84 (0.83–0.86)	0.81 (0.80–0.82)	0.62
	Procedure Codes	0.86 (0.84–0.87)	0.76 (0.75–0.77)	0.36
	Diagnosis Codes	0.95 (0.94–0.96)	0.91 (0.90–0.92)	0.25
	Procedure + Diagnosis Codes	0.96 (0.95–0.96)	0.92 (0.91–0.92)	0.33

*Out-of-Sample estimates were calculated using non-matched records

**PTOS = Pennsylvania Trauma Outcomes Study

## Discussion

We found that claims-based models using Medicare data provide a good approximation for the risk adjustment approach that can be achieved through registry data. The variables in the claims-based models include many of the categories found to be important in the original Trauma Quality Improvement Project paper, namely patient demographics, comorbidities and key characteristics of the injury (i.e., mechanism, severity, body region impacted) [10,12]. The AUC for the demographics and clinical risk factors model exceeded 0.80 for both the matched and out-of-sample cohorts, while correlation with the gold-standard registry model exceeded 0.60. Using claims to estimate the probability of death after injury allows for a more generalizable method by which to benchmark trauma center performance. More importantly, it represents a novel approach for benchmarking outcomes at non-trauma center hospitals in order to capture an accurate representation of regional injury outcomes [1].

We also explored the use of the top 500 procedure and diagnostic codes in improving our ability to predict mortality. Inclusion of diagnostic codes improved the AUC to >0.9 for all samples. However, it led to a decreased correlation with probability of death deduced from registry data. We note that diagnosis and procedure codes include information about care delivered after the patient reaches the hospital: it is conceivable that inclusion of this information may overestimate the probability of death in comparison to models that only use information available at the time of the injury or hospital arrival. The goal of the risk adjustment model is not to simply predict death, but to allow for measurement of outcomes after accounting for differences in a patient population. Additionally, the risk adjustment model is not intended to penalize hospitals that treat more ill or more severely injured patients. Consequently, the inclusion of diagnosis and procedure codes may be overcorrecting for the baseline severity, accounting instead for the complications or subsequent care needed throughout the hospitalization.

Analogous to GLM, LASSO models are easy to interpret because features are combined linearly and weights are assigned to each feature (S1 and S2 Tables). LASSO modeling is an innovative approach to risk adjustment and represents an advance over earlier GLM or traditional logistic regression models. Specifically, LASSO’s L1 regularization technique allows for weights to be zero, resulting in dimensionality reduction. In turn, this helps discard features when dealing with a large number of claims-based variables [15–17]. LASSO’s dimensionality reduction specifically helped to adjust for the hundreds of CMS attributes considered in our claims-based models, including demographics, clinical risk factors, and diagnosis and procedure codes. Unchanged, this same method can be applied to risk adjustment efforts that rely on other large data sources. Additionally, it could prove especially important for conditions with no national registry, such as sepsis or cardiac arrest, where generalizability of existing data is a concern.

Our use of administrative data introduced a number of analytical limitations that require discussion. First, by necessity, we relied on probabilistic matching to create a dataset for model building and performance assessment. Despite our use of stringent filtering criteria throughout, it remains formally possible, the PTOS and Medicare linkages may be incorrect for some of the analyzed patients. In light of this possibility, we gauged the generalizability of our models by using both matched and out-of-sample cohorts. Second, our analyses used only those variables that Medicare collects for billing purposes. Information about the timing and location of the injury, or the specifics of the injury itself, are not readily available. Because of this, all injury characteristics were derived. Validated measures were used for these variables, but information may not be complete or accurate given differences in coding practices among hospitals, and the limited number of fields available for reporting. Similarly, this lack of granularity extends to clinical confounders and administrative data may not capture all treatments, interventions, and laboratory. Our models looking at the top diagnoses and procedure codes were an attempt to include as many of the captured codes as possible and evaluate their contribution to claims-based risk adjustment models.

We also note that it may be valuable to perform risk adjustment in younger patients without having to rely on trauma registry data. Similarly, our analysis focused on trauma centers in Pennsylvania. but multi-state or national models may be needed. In both instances, the modeling approach described can be applied to other administrative data sources, as well as Medicare data from trauma centers and non-trauma hospitals across the country. Subsequent validation and calibration work should be completed in the case of non-trauma centers and for models including younger patients, and additional years beyond 2013–2014 may also be warranted. Nonetheless, it will be possible to create general models easily given that the same variables are available across all hospitals that serve Medicare patients, and that similar data and coding is used in numerous other data sources captured for billing purposes.

## Methods

### Data sources and population

We used two data sources. One was the Pennsylvania Trauma Outcomes Study (PTOS) registry data from the Pennsylvania Trauma Systems Foundation, which are the type of data used for conventional trauma center benchmarking. The second source was CMS claims data for hospitals in Pennsylvania, including inpatient stays from the Medicare Provider Analysis and Review (MedPAR) Research Identifiable File (RIF), outpatient visits from the Outpatient claims RIF, and demographic, geographic, date-of-death, and enrollment information from the Master Beneficiary Summary File (Base Segment) and Vital Status File. All injury-related patients from the PTOS and CMS data were included for analysis if they were ≥65 years and treated in a Pennsylvania hospital in 2013 or 2014. In the CMS data, ICD-9-CM codes were used to identify injury as a primary or secondary diagnosis, (ICD9-CM 800–959 (injuries) excluding 905–909 (late effects of injury), 930–939 (foreign bodies), or 958 (complications of injury), as defined by the National Center for Injury Prevention and Control).

### Feature engineering

For each dataset, admission date and date of birth were divided into month, day, and year components to facilitate matching. Naming conventions for Race/Ethnicity data were standardized across data sources. In-hospital death in CMS data was derived from the Vital Status file, and a beneficiary was coded as deceased if they had a valid date of death on or before the date of discharge, regardless of the disposition code. Injury severity scores (ISS), Abbreviated Injury Scores (AIS), and maximum AIS by body region in claims data were calculated using ICD-9 and E-Codes via the *icdpicr* (version 1.0.0) package [18]. In registry data, age, systolic blood pressure, and pulse rates were categorized into seven percentiles to allow for easier interpretability and more parsimonious models.

### Matching patients across data sources

In order to compare risk adjustment between claims data and the gold standard registry data, we created a matched cohort comprised of patients whose records could be identified in the two data sources, Medicare claims and PTOS. All trauma cases in the PTOS registry were eligible for the matching process if they met age criteria (≥65 years) and contained values for the following attributes: sex, birth date, injury date, race/ethnicity, residential zip code, and hospital ID. Trauma cases in the CMS data were deemed eligible for the matching process if they met the inclusion and exclusion criteria of the PTOS registry for the year of their claim [19]. To increase the match rate and validate matches between the two datasets, we requested permission from Pennsylvania trauma centers to use their hospital identity.

### Linking claims and registry records

#### Facility to facility matching

To improve the quality of matches, we first performed pairwise comparisons between all facilities in the PTOS and CMS datasets using deidentified hospital facility identifiers. For each facility pair, we calculated two metrics:

1. CMS cohort metric: each individual Medicare trauma case in the candidate facility was given the maximal score when compared against each PTOS case in the candidate facility. One point was earned for matching each attribute (sex, residential zip code, year of birth, month of birth, day of birth, year of admission, month of admission, day of admission, and a combined race/ethnicity value). The mean score across all Medicare cases for the candidate facility was used as the CMS cohort metric.
2. PTOS cohort metric: because the number of cases in the CMS and PTOS cohorts differed, we calculated a similar metric in the opposite direction. Specifically, each individual PTOS case in the candidate facility was given the maximal score when compared against each CMS case in the candidate facility using the same point system noted above. The mean score across all PTOS cases for the candidate facility was used as the PTOS cohort metric.

For each CMS facility, the compared PTOS facilities were ranked in decreasing order of the CMS cohort metric. Similarly, for each PTOS facility, the compared CMS facilities were ranked in decreasing order of the PTOS cohort metric. A facility was deemed a match only if both the CMS and PTOS cohort metrics were top-ranked for the pair. Manual validation of the matched facilities occurred using known facility pairs from the subset of facilities that provided permission.

### Matching claims and registry records within facilities

Within each matched facility, a similar process was used to identify cases whose Medicare record could be linked to their PTOS record. Using the same attributes as in the preceding section, pairwise comparisons were performed between CMS and PTOS records within the matched facility, earning one point for each attribute. Records were considered linked only if all of the following conditions were met: (i) there was no more than one mismatched attribute, (ii) for either patient in the pair, there was no other patient it paired with that scored the same or better, and (iii) the patient outcome (died vs survived) status agreed between the CMS and PTOS datasets.

### Model development

All models used in-hospital death (survival to discharge) as the outcome. Two registry-based models were estimated using demographics, injury characteristics, comorbidities [10,12], and clinical characteristics to create a parsimonious model: model 1 was estimated using Generalized linear models (GLM); and model 2 was estimated using Least absolute shrinkage and selection operator (LASSO) (**Table 3**) [15,20]. For all claims-based models, LASSO was used given the large number of clinical features.

**Table 3:** Variables of interest for each trauma risk adjustment model.

Variable	PTOS Data	Medicare Claims Data				
	Registry LASSO Model	Demographics	Demographics + Clinical Risk Factors	Procedure Codes	Diagnosis Codes	Procedure + Diagnosis Codes
Age	Categorized into 7 percentiles	Continuous	Continuous			
Female	Male/Female	Male/Female	Male/Female			
Race		White/Black/ Other	White/Black/ Other			
Transferred	Yes/No		Yes/No			
Skilled Nursing Facility Resident			Yes/No			
Year of Injury/Admission			2013/2014			
Abbreviated Injury Score	0–6, Unknown		0–6, Unknown			
Maximum Score, Overall						
Maximum Score–Head	0–6, Unknown		0–6, Unknown			
Maximum Score–Face			0–6, Unknown			
Maximum Score–Chest			0–6, Unknown			
Maximum Score–Abdomen			0–6, Unknown			
Maximum Score–Extremities			0–6, Unknown			
Maximum Score–External			0–6, Unknown			
Injury Severity Score			1–75, Unknown			
Mechanism of Injury	Fall, Fire/Burn, Pedal Cyclist, Motor Vehicle, Other or Unknown		Fall, Fire/Burn, Pedal Cyclist, Motor Vehicle, Other or Unknown			
External Cause of Injury Code			Primary ECode			
Systolic Blood Pressure	Categorized into 7 percentiles		Shock			
Pulse	Categorized into 7 percentiles		Tachycardia			
Cardiac Arrest	Systolic BP <90 Yes/No		ICD-9-CM Code 427.5 primary diagnosis: Yes/No			
Glasgow Coma Score	Categorized Score		Altered Mental Status; Impaired Sensorium			
Comorbidities	Diabetes, Peripheral Vascular Disease		All Elixhauser Comorbidities			
Procedure Codes (CMS)				Up to 500		Up to 500
Diagnosis Codes (CMS)					Up to 500	Up to 500

Models were generated using matched patients. They were also evaluated against unmatched (out-of-sample) cases to gauge their generalizability against unseen outcomes (**Fig 1**). To avoid data leakage and overfitting, no cases evaluated in the unmatched cohort were part of the matched training set used during model creation. Ninety-five percent confidence intervals (CI) were computed using 2000 stratified bootstrap replicates. All analyses were conducted using R version 3.6.3 (R Foundation for Statistical Computing, Vienna, Austria). GLMs and the LASSO models were created using the *stats* and *glmnet* packages, respectively [15]. For LASSO, 10-fold cross-validation was used to choose the optimal lambda value. For both the GLM and LASSO models, CI for the area under the curve (AUC) were calculated using the *pROC* version 1.16.2 package [21]. Injury characteristics in claims data were calculated using the *icdpicr* (version 1.0.0) package [18].

Table 3 lists the variables used across the registry and claims-based models. Registry models included previously identified variables [10,12]. For claims-based models, variables were selected for inclusion if they were an exact match for those in TQIP (age, sex, single worst Abbreviated Injury Score, mechanism of injury, transfer status), or if they were the billing code equivalent of the physiologic factors captured in registry data (impaired sensorium, tachycardia, altered mental status, shock). Additional comorbidities, patient demographics, clinical characteristics, and the 500 most frequent diagnosis and procedure codes were also considered. Variables were then grouped into three categories: demographics, procedure and diagnosis codes, and clinical risk factors.

### Correlations using patient-level probability of death

After fitting each model outlined above, the probability of death was estimated for each patient in the matched cohort. For out-of-sample testing, the same models were used to predict death outcomes for the eligible but unmatched cohort. The AUC was calculated for each model, and separately for each out-of-sample evaluation, to estimate the models’ ability to predict a death outcome. To avoid data leakage when calculating the out-of-sample AUC, the models were created using only the matched cases with no overlap with the unmatched cohort. For comparison between each of the claims-based models and the registry model, we calculated the Spearman rank correlation coefficient using the patient-level outcome probabilities for the matched patients between each model pair.

**Meeting Presentations:** A portion of this work was presented at the 2020 American Association for the Surgery of Trauma Annual Meeting and Clinical Congress of Acute Care Surgery and the 2020 European Congress of Trauma and Emergency Surgery (cancelled due to COVID-19).

## Supporting information

S1 Table Comparison of model coefficients for GLM and LASSO models using Pennsylvania Trauma Outcomes Study (PTOS) registry data. (DOCX)

S2 Table Comparison of model coefficients for LASSO models using Centers for Medicare and Medicaid Services Medicare claims data. (DOCX)

## References

[1] National Academies of Sciences, Engineering, and Medicine. A National Trauma Care System: Integrating Military and Civilian Trauma Systems to Achieve Zero Preventable Deaths After Injury. Washington, DC: The National Academies Press. 2016. 10.17226/23511. 27748086

[2] Sise R, Calvo R, Spain D, Weiser T, Staudenmayer K. The epidemiology of trauma-related mortality in the United States from 2002 to 2010. J Trauma Acute Care Surg. 2014;76(4):913–9; discussion 920. doi: 10.1097/TA.0000000000000169 24662852

[3] Corso P, Finkelstein E, Miller T, Fiebelkorn I, Zaloshnja E. Incidence and lifetime costs of injuries in the United States. Inj Prev. 2006;12(4):212–8. doi: 10.1136/ip.2005.010983 16887941 PMC2586784

[4] Centers for Disease Control and Prevention. Injury Prevention & Control: Data & Statistics (WISQARSTM: Web-based Injury Statistics Query and Reporting System). Centers for Disease Control and Prevention. 2015.

[5] American College of Surgeons—Committee on Trauma. Resources for optimal care of the injured patient: 2022 Standards. Chicago, IL: American College of Surgeons; 2022.

[6] Demetriades D, Martin M, Salim A, Rhee P, Brown C, Doucet J, et al. Relationship between American College of Surgeons trauma center designation and mortality in patients with severe trauma (injury severity score > 15). J Am Coll Surg. 2006;202(2):212–5; quiz A45. 16427544 10.1016/j.jamcollsurg.2005.09.027

[7] MacKenzie E, Rivara F, Jurkovich G, Nathens A, Frey K, Egleston B, et al. A national evaluation of the effect of trauma-center care on mortality. N Engl J Med. 2006;354(4):366–78. doi: 10.1056/NEJMsa052049 16436768

[8] Delgado M, Yokell M, Staudenmayer K, Spain D, Hernandez-Boussard T, Wang N. Factors associated with the disposition of severely injured patients initially seen at non–trauma center emergency departments: disparities by insurance status: Disparities by insurance status. JAMA Surg. 2014;149(5):422–30. 24554059 10.1001/jamasurg.2013.4398 PMC4422057

[9] Xiang H, Wheeler K, Groner J, Shi J, Haley K. Undertriage of major trauma patients in the US emergency departments. Am J Emerg Med. 2014;32(9):997–1004. doi: 10.1016/j.ajem.2014.05.038 24993680

[10] Newgard C, Fildes J, Wu L, Hemmila M, Burd R, Neal M, et al. Methodology and analytic rationale for the American College of Surgeons Trauma Quality Improvement Program. J Am Coll Surg. 2013;216(1):147–57. doi: 10.1016/j.jamcollsurg.2012.08.017 23062519

[11] The National Association of State EMS Officials. Status of State Trauma System Planning and Development. The National Association of State EMS Officials. 2016. Available from: https://www.nasemso.org/Resources/Monographs/documents/Status-of-State-Trauma-System-Planning-and-Development-Sept2016.pdf.

[12] Wiebe D, Holena D, Delgado M, McWilliams N, Altenburg J, Carr B. The Pennsylvania trauma outcomes study risk-adjusted mortality model: Results of a statewide benchmarking program. Am Surg. 2017;83(5):445–52. 28541852 PMC5852669

[13] National Quality Forum. Population Based Trauma Outcomes. NQF. 2019. Available from: https://www.qualityforum.org/Publications/2019/05/Trauma_Outcomes_Final_Report.aspx

[14] Velopulos C, Enwerem N, Obirieze A, Hui X, Hashmi Z, Scott V, et al. National cost of trauma care by payer status. J Surg Res. 2013;184(1):444–9. doi: 10.1016/j.jss.2013.05.068 23800441 PMC5995319

[15] Friedman J, Hastie T, Tibshirani R. Regularization Paths for Generalized Linear Models via Coordinate Descent. J Stat Softw. 2010;33(1):1–22. doi: 10.1109/TPAMI.2005.127 20808728 PMC2929880

[16] Loher P, Karathanasis N. Machine Learning Approaches Identify Genes Containing Spatial Information From Single-Cell Transcriptomics Data. Front Genet. 2020;11:612840. doi: 10.3389/fgene.2020.612840 33633771 PMC7902049

[17] James G, Witen D, Hastie T, Tibshirani R. An introduction to statistical learning with applications in R. 1 ed. New York, NY: Springer; 2013.

[18] Clark D, Black A, Skavdahl D, Hallagan L. Open-access programs for injury categorization using ICD-9 or ICD-10. Inj Epidemiol. 2018;5(1):11. doi: 10.1186/s40621-018-0149-8 29629480 PMC5890002

[19] Operational Manual for the Data Base Collection System. Mechanicsburg, PA: Pennsylvania Trauma Systems Foundation; 2015.

[20] Tibshirani R. Regression shrinkage and selection via the lasso. J R Stat Soc. 1996;58(1):267–88.

[21] Robin X, Turck N, Hainard A, Tiberti N, Lisacek F, Sanchez JC, et al. pROC: an open-source package for R and S+ to analyze and compare ROC curves. BMC Bioinformatics. 2011;12(1):77. doi: 10.1186/1471-2105-12-77 21414208 PMC3068975

