# Supplementary material for: Using medicare claims to estimate risk-adjusted performance of Pennsylvania trauma centers
Source: PLOS Digit Health. 2023 Jun 2;2(6):e0000263. doi: 10.1371/journal.pdig.0000263 (PMC10237397; doi:10.1371/journal.pdig.0000263)
Supplement: S1 Table — (DOCX) [file pdig.0000263.s001.docx]

**S1 Table. Comparison of model coefficients for GLM and Lasso using registry data**

| **Variable** | **PTOS Data** | |
| --- | --- | --- |
|  | **Registry Model (GLM)** | **Registry Model (Lasso)** |
| (Intercept) | -1.57 | -1.32 |
| Age | 0.21 | 0.15 |
| Female | -0.39 | -0.31 |
| Transfer | -0.11 |  |
| Arrest Systolic BP <90 | 1.77 | 1.79 |
| Diabetes | 0.41 |  |
| Peripheral Vascular Disease | 0.63 | 0.36 |
| Glasgow Coma Score | | |
| 2 | -0.07 | 0 |
| 3 | 0.06 | 0.04 |
| 4 | -0.54 | -0.16 |
| 5 | -1.57 | -1.16 |
| 6 | -2.81 | -2.55 |
| No score recorded | -2.75 | -2.29 |
| Pulse Rate (seven categories) |  |  |
| 1 | -0.25 | -0.05 |
| 2 | -0.18 | -0.01 |
| 3 | -0.03 |  |
| 4 | 0.01 |  |
| 5 | 0.05 |  |
| 6 | 0.52 | 0.47 |
| No pulse recorded | 0.14 |  |
| Systolic Blood Pressure (seven categories) |  |  |
| 1 | -0.53 |  |
| 2 | -0.52 |  |
| 3 | -0.76 | -0.12 |
| 4 | -0.73 | -0.11 |
| 5 | -0.80 | -0.16 |
| 6 | -0.76 | -0.11 |
| No systolic blood pressure recorded | 0.45 | 0.28 |
| Maximum AIS - Head | | |
| 1 | 0.20 |  |
| 2 | 0.26 |  |
| 3 | 0.38 | 0.22 |
| 4 | 0.01 |  |
| 5 | 0.79 | 0.39 |
| 6 | 13.44 |  |
| Maximum AIS by Body Region | | |
| Face | 0.03 | -0.52 |
| Thorax | 0.93 | 0.18 |
| Abdomen | 1.44 | 0.57 |
| Extremities | 2.16 | 1.66 |
| External | 3.86 | 2.76 |
| No region recorded | 2.67 | 1.52 |
| Mechanism of Injury | | |
| Drowning/submersion | -12.12 |  |
| Fall | 0.09 |  |
| Fire/burn | 1.58 | 0.87 |
| Firearm | 1.23 | 0.98 |
| Machinery | 0.18 |  |
| Motor vehicle/traffic | 0.50 | 0.37 |
| Natural/environmental | -13.12 |  |
| Overexertion | -12.58 |  |
| Pedal/cyclist | -1.49 | -0.18 |
| Pedestrian | 1.62 | 0.61 |
| Struck-by/against | -1.14 | -0.37 |
| Suffocation | -12.10 |  |
| Transport | -0.30 |  |
| Unspecified | -0.28 |  |
| No mechanism reported | -0.52 |  |
