# Supplementary material for: Using medicare claims to estimate risk-adjusted performance of Pennsylvania trauma centers
Source: PLOS Digit Health. 2023 Jun 2;2(6):e0000263. doi: 10.1371/journal.pdig.0000263 (PMC10237397; doi:10.1371/journal.pdig.0000263)
Supplement: S2 Table — (DOCX) [file pdig.0000263.s002.docx]

**S2 Table. Comparison of model coefficients for Lasso models using Medicare data**

|  | **Medicare Claims-Based Model** | | | | | | |
| --- | --- | --- | --- | --- | --- | --- | --- |
| **Variable** | **Demographics** | **Demographics + Clinical Risk Factors** |  | **Variable** | **Procedure Codes** | **Diagnosis Codes** | **Procedure + Diagnosis Codes** |
| (Intercept) | -2.73 | -5.73 |  | (Intercept) | -3.54 | -3.87 | -4.13 |
| Age | -0.35 | 0.03 |  | Procedure Codes |  |  |  |
| Female | 0.00 | -0.27 |  | 110 | 0.20 |  |  |
| White |  | 0.08 |  | 124 | 0.32 |  |  |
| Transfer |  | -1.03 |  | 125 | 0.62 |  |  |
| Cardiac Arrest |  | 3.70 |  | 131 | 0.62 |  |  |
| Altered Mental Status |  | 0.35 |  | 139 | 0.60 |  |  |
| Shock |  | 1.03 |  | 17 | 0.70 |  | 0.51 |
| Congestive Heart Failure |  | 0.18 |  | 180.9 | 0.89 |  |  |
| Hypothyroidism |  | -0.14 |  | 212 | 0.78 |  |  |
| Liver Disease |  | 0.30 |  | 221 | 0.33 |  |  |
| Metastatic Cancer |  | 0.36 |  | 311 | -1.00 |  | -0.16 |
| Cardiac Arrhythmias |  | 0.16 |  | 315.00 | 0.19 |  |  |
| Coagulopathy |  | 0.25 |  | 325.51 | 1.45 |  | 0.17 |
| Fluid and Electrolyte Disorders |  | 0.14 |  | 365.56 | 1.47 |  | 1.30 |
| Valvular Disease |  | -0.12 |  | 379.1 | 0.96 |  | 0.79 |
| Depression |  | -0.16 |  | 389.1 | 0.76 |  | 0.55 |
| Hypertension |  | -0.29 |  | 389.3 | 0.40 |  | 0.16 |
| Paralysis |  | 0.42 |  | 389.5 | 0.19 |  |  |
| Neurological Disorders, Other |  | 0.23 |  | 431.1 | -0.88 |  | -0.34 |
| Maximum AIS - Head |  |  |  | 459.1 | 1.32 |  | 0.78 |
| 2 |  | -0.28 |  | 506.1 | 0.52 |  | 0.04 |
| 3 |  | 0.27 |  | 517.02 | 0.16 |  |  |
| 5 |  | 2.42 |  | 541.1 | 0.37 |  | 0.41 |
| 6 |  | 0.40 |  | 541.9 | 0.85 |  |  |
| Maximum AIS by Body Region |  |  |  | 793.2 | -0.08 |  |  |
| Head & Neck |  | 0.24 |  | 793.6 | -0.11 |  |  |
| Abdomen |  | 0.06 |  | 870.3 | 0.11 |  |  |
| Extremities |  | -0.04 |  | 929.50 | 3.43 |  | 2.75 |
| Mechanism of Injury |  |  |  | 939.0 | 0.28 |  | 0.01 |
| Firearm |  | 1.15 |  | 960.4 | 0.32 |  | 0.53 |
| Pedestrian |  | 0.44 |  | 966 | 0.03 |  |  |
| Injury Severity Score |  | 0.02 |  | 967.1 | 2.62 |  | 1.47 |
| External Cause of Injury Codes |  |  |  | 967.2 | 1.48 |  | 0.07 |
| E810.1 |  | 0.29 |  | 990.5 | 0.30 |  |  |
| E814.7 |  | 0.06 |  | 992.91 | 0.93 |  | 0.81 |
| E814.8 |  | 1.45 |  | 996.0 | 2.28 |  | 0.92 |
| E815.1 |  | 0.05 |  | 996.2 | 0.96 |  | 0.68 |
| E815.7 |  | 2.28 |  | Diagnosis Codes |  |  |  |
| E819.2 |  | 0.08 |  | 263.9 |  | -0.04 |  |
| E822.7 |  | 0.50 |  | 272.4 |  | -0.23 |  |
| E825.9 |  | 2.70 |  | 275.2 |  | -0.28 |  |
| E848 |  | 0.12 |  | 276.0 |  | 0.15 | 0.05 |
| E879.8 |  | 0.39 |  | 276.1 |  | -0.06 |  |
| E880.9 |  | 0.06 |  | 276.2 |  | 0.62 | 0.42 |
| E885.9 |  | -0.29 |  | 276.8 |  | -0.15 |  |
| E893.8 |  | 0.68 |  | 285.1 |  | -0.28 |  |
| E893.9 |  | 2.41 |  | 286.9 |  | 0.86 | 0.18 |
| E925.1 |  | -1.95 |  | 305.1 |  | -0.06 |  |
| E939.4 |  | 0.61 |  | 310.2 |  | -0.09 |  |
| E955.0 |  | 1.54 |  | 348.4 |  | 2.22 | 1.55 |
| E955.4 |  | 1.43 |  | 348.5 |  | 0.96 | 0.71 |
| E955.9 |  | 1.49 |  | 349.82 |  | -0.05 |  |
| E957.1 |  | 3.42 |  | 389 |  | 0.49 | 0.33 |
| E988.9 |  | 1.00 |  | 401.0 |  | -0.00 |  |
|  |  |  |  | 401.9 |  | -0.14 |  |
|  |  |  |  | 410.71 |  | 0.04 |  |
|  |  |  |  | 415.19 |  | 0.01 |  |
|  |  |  |  | 427.5 |  | 3.34 | 2.37 |
|  |  |  |  | 434.11 |  | 0.46 |  |
|  |  |  |  | 458.9 |  | 0.15 |  |
|  |  |  |  | 518.51 |  | 1.34 | 0.41 |
|  |  |  |  | 518.52 |  | 0.33 |  |
|  |  |  |  | 518.81 |  | 1.76 | 0.79 |
|  |  |  |  | 518.84 |  | 0.76 |  |
|  |  |  |  | 530.81 |  | -0.03 |  |
|  |  |  |  | 564.00 |  | -0.54 | -0.01 |
|  |  |  |  | 571.5 |  | 0.07 |  |
|  |  |  |  | 584.9 |  | 0.12 |  |
|  |  |  |  | 599.0 |  | -0.28 |  |
|  |  |  |  | 707.22 |  | -0.46 |  |
|  |  |  |  | 780.2 |  | -2.04E-05 |  |
|  |  |  |  | 780.97 |  | 0.37 |  |
|  |  |  |  | 781.2 |  | -0.45 |  |
|  |  |  |  | 785.52 |  | 0.67 | 0.09 |
|  |  |  |  | 786.09 |  | 0.15 |  |
|  |  |  |  | 787.20 |  | -0.25 |  |
|  |  |  |  | 788.20 |  | -0.11 |  |
|  |  |  |  | 799.3 |  | -0.18 |  |
|  |  |  |  | 801.21 |  | 0.00 |  |
|  |  |  |  | 801.26 |  | 0.58 |  |
|  |  |  |  | 805.04 |  | 0.28 |  |
|  |  |  |  | 805.06 |  | 0.12 |  |
|  |  |  |  | 813.42 |  | -0.09 |  |
|  |  |  |  | 852.25 |  | 2.31 | 1.32 |
|  |  |  |  | 853.00 |  | 0.05 |  |
|  |  |  |  | 864.05 |  | 0.76 | 0.21 |
|  |  |  |  | 868.04 |  | 0.40 |  |
|  |  |  |  | 958.4 |  | 1.42 | 0.88 |
|  |  |  |  | 995.91 |  | 0.07 |  |
|  |  |  |  | E8809 |  | 0.59 |  |
|  |  |  |  | E8889 |  | 0.37 |  |
|  |  |  |  | V1255 |  | -0.04 |  |
|  |  |  |  | V1582 |  | -0.24 |  |
|  |  |  |  | V481 |  | -0.01 |  |
|  |  |  |  | V4986 |  | 1.03 | 0.88 |
|  |  |  |  | V667 |  | 2.47 | 2.38 |
